# Croatian Models and Experience in First-Episode Psychosis Treatment

**DOI:** 10.1192/j.eurpsy.2022.87

**Published:** 2022-09-01

**Authors:** A. Savic, D. Ostojic, P. Brecic

**Affiliations:** 1 University Psychiatric Hospital Vrapce, Department Of Diagnostics And Intensive Care, First-episode Psychosis Unit, Zagreb, Croatia; 2 University Psychiatric Hospital Vrapce, Department Of Affective Disorders, Zagreb, Croatia

**Keywords:** early intervention, First-episode psychosis, service integration, schizophrénia

## Abstract

We have witnessed a significant push towards staging in medicine. That trend has not bypassed psychiatry, with realization that early phases of various disorders present the window for early intervention that is most likely to result in preserving every-day functionality and achieving favourable outcomes. First-episode psychosis programs have been developed in order to ensure adequate early interaction with psychiatric services, help achieve faster and quality remission, prevent relapses and ensure better long-term outcomes. There is still, however, no consensus on the format or the most appropriate intervention in the early-course psychosis. Patients in Zagreb, Croatia, are offered a number of first-episode psychosis programs, one of which is housed in the largest Croatian psychiatric institution, University Psychiatric Hospital Vrapce. Specialized early-course treatment model in Vrapce stemmed from first-episode inpatient unit established in 2004, and grew to present in its core integration of care across different organizational units, acuity levels, and specific patient needs. Recognizing that a significant number of first-episode patients first interact with psychiatric services through emergency units, Vrapce’s model fostered early interaction with specialized services staff starting with intensive and emergency care units, allowing for continuity of care and early recruitment into specialized services. Vertical integration meant inpatient acute and subacute units seamlessly linked with day hospital and outpatient services, creating the setting for earlier formation of therapeutic alliance and treatment plans, but also allowing for flexible entry points for users. COVID-19 pandemic, in addition to challenging the initial integrations of services, facilitated transfer of certain services into virtual space.

**Disclosure:**

No significant relationships.

